# We-making through technology: social cohesion and virtual adaptation in community arts organizations for artists with IDD during the COVID-19 pandemic

**DOI:** 10.3389/fpubh.2026.1848823

**Published:** 2026-07-10

**Authors:** Yumi Shirai, Michelle Boulanger Thompson, Tamara Shetron

**Affiliations:** 1 Department of Family and Community Medicine, Sonoran University Center for Excellence in Disabilities, University of Arizona, Tucson, AZ, United States; 2 Department of Occupational Therapy, School of Rehabilitation Sciences, Old Dominion University, Norfolk, VA, United States; 3 College of Education, Texas State University, San Marcos, TX, United States

**Keywords:** community-based, COVID-19 pandemic, creative arts, intellectual and/or developmental disabilities, social connection and belonging, technology, virtual, we-making

## Abstract

COVID-19 public health restrictions disrupted in-person arts participation, increasing risks of social isolation and reduced community access. Prior research shows that many people engaged in home-based arts and cultural activities during lockdown, supporting emotional regulation, a sense of belonging, and social connection; however, little is known about how these shifts affected community arts organizations that serve artists with intellectual and/or developmental disabilities (IDD), a population disproportionately impacted by the pandemic and longstanding inequities. We examined how community-based arts organizations adapted programming for artists with IDD during the COVID-19 pandemic, including barriers and unanticipated outcomes relevant to equitable access and participation in community living. We conducted an online survey (*n* = 28) and semi-structured interviews (*n* = 25) with representatives of community-based creative organizations. Descriptive statistics and thematic analysis characterized technology use and adaptation strategies, and findings were interpreted using the We-Making framework. Technology use primarily emphasized connection (96%) and access (96%), with comparatively less emphasis on inclusion (79%), visibility (63%), and creativity (45%). Four roles of technology were identified: (1) enabling social connection, (2) serving as a temporary and often suboptimal substitute for in-person engagement, (3) catalyzing innovation, and (4) driving pedagogical change. Rapid implementation created new pathways for participation while also exposing persistent access barriers and prompting reflection on equitable practice. Digital and hybrid adaptations supported continuity of engagement for some artists with IDD and created opportunities to reassess program design. These findings highlight community arts organizations as public health partners for inclusive, accessible approaches to social connection and wellbeing beyond the immediate responses to crises and recovery.

## 1 Introduction

The COVID-19 pandemic disrupted in-person arts and cultural participation while increasing population-level needs for coping resources and social connection. Evidence indicates that during lockdown, many people engaged in home-based arts and cultural activities as (1) a way to manage emotions and support self-growth during heightened stress and uncertainty (1), (2) a method to foster a sense of belonging (2), and (3) a source of social support and connection (3). In response, arts organizations quickly innovated through digital and hybrid programming, which became a “lifeline” for some groups, especially when coordinated with health and social care partnerships (4).

Community-based arts organizations serving artists with intellectual and/or developmental disabilities (IDD) faced similar disruptions, including the suspension of in-person services following public health mandates. These organizations, including visual art studios, galleries, theater and performing arts groups, and recreational programs, support routine, social connection, and creative expression for artists with IDD. Service disruptions, therefore, posed increased risks for artists who already face disproportionate barriers to health and mental health care and higher rates of anxiety, stress, and social isolation (5). The pandemic intensified these challenges through the loss of community access, resources, and familiar routines (6), with reported effects including increased worry, less sleep, more screen time, and a greater need for help with daily tasks (7). Adults with IDD also faced higher risks of COVID-19 infection, hospitalization, and death (5, 8, 27).

Meanwhile, community arts participation can be a vital contributor to wellbeing. Access to arts and creative expression offers opportunities for self-expression, relationship building, and social involvement. Successful community-based creative programs often emphasize process over product, foster cohesion and relationship-building, provide sensory and emotional gratification, and offer supportive structures (9). These settings may also create inclusive community spaces that foster belonging and resilience, particularly for people with IDD (10, 11) and support the development of artistic, interpersonal, and professional skills (12, 13). By bringing together people with and without disabilities as collaborators, community-based art groups can also challenge norms and promote innovation and inclusive meaning-making (14).

Technology has traditionally been viewed as assistive technology to support participation, inclusion, and accessibility for people with disabilities (15, 16). However, common digital tools are now integrated into everyday life and increasingly facilitate social contact and engagement in meaningful activities beyond just assistive functions (17). During the pandemic, these tools became especially important for maintaining participation and reducing loneliness and isolation, with potential benefits for health and wellbeing (18). Nevertheless, rapid adoption of virtual platforms also raises concerns about equity, accessibility, and the quality of participation, especially for artists with IDD and the organizations that support them.

This mixed-methods study examined how community-based arts organizations serving artists with IDD shifted to online programming during the COVID-19 pandemic and what these adaptations meant for equitable participation.

**Figure 1:**
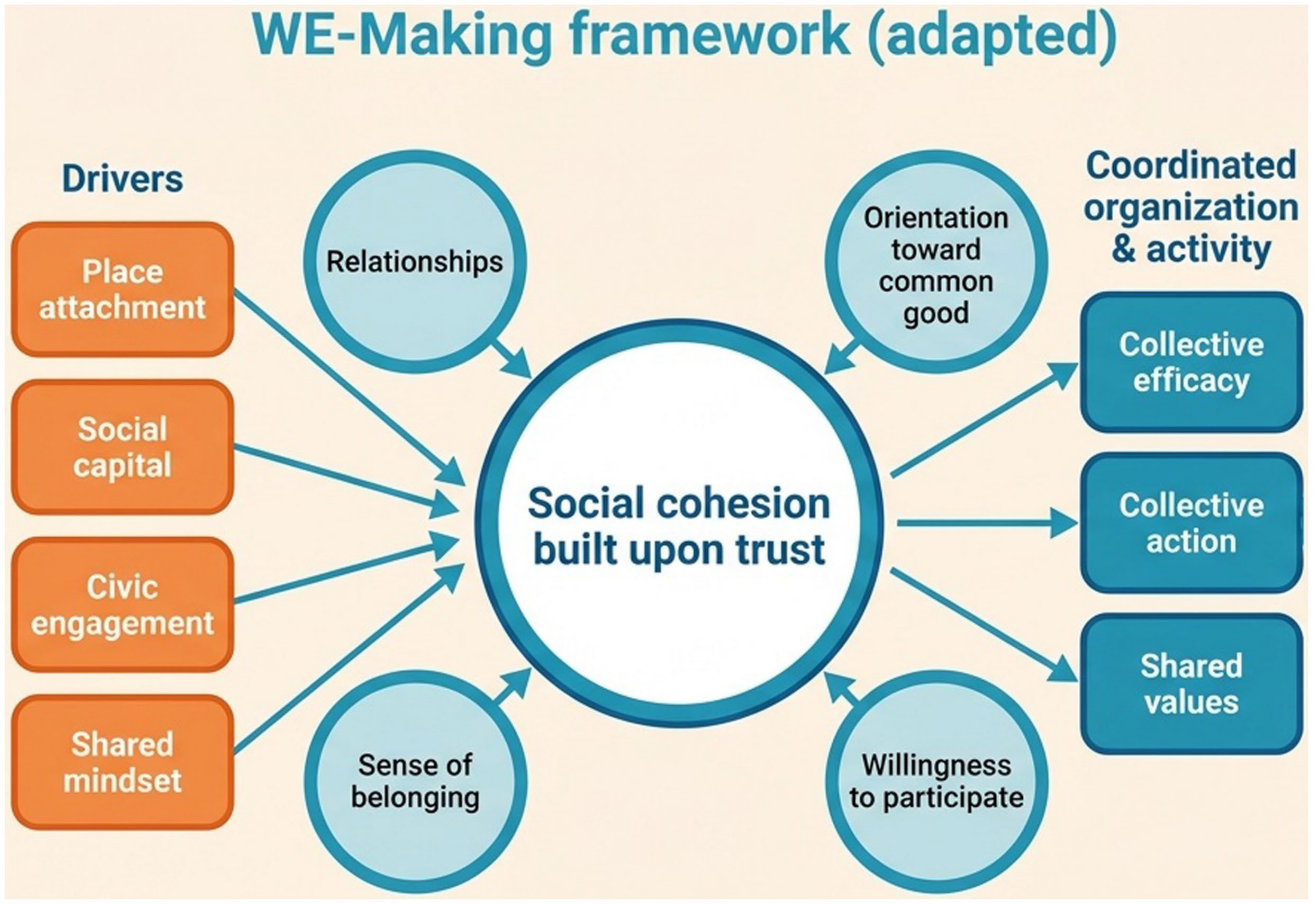
Key elements of the We-Making framework for supporting social cohesion and leading coordinated, equitable change. Adapted in part from the WE-Making conceptual framework (19). Adapted with permission. Source: https://metrisarts.com/wp-content/uploads/2021/04/we-making_literature-review.pdf.

The We-Making Framework, an evidence-informed framework, illustrates how community-based arts and cultural spaces promote social cohesion and equitable wellbeing in community settings (19). The framework defines social cohesion as when “individuals feel and act as part of a group oriented toward working together” (p. 21), grounded in five characteristics: trust, relationships, sense of belonging, willingness to participate, and orientation toward the common good for equitable change. Please see the key components of the We-Making Framework (19) in Figure 1. The We-Making Framework adopts a broad definition of transient communities, including temporary, resettled, dispersed, displaced, and online communities, in theory. It has been used in a systematic review identifying evidence supporting this theoretical model, although without any online programs (20). This study contributes empirical data to help confirm elements of the framework in an online setting. The framework is useful for examining the adaptations these creative organizations made, particularly when using the virtual environment as a newly shared community space during the unique historical event of the COVID-19 pandemic.

Our research questions were guided by three assumptions: (a) community-based creative arts organizations can promote creativity, visibility, access, connection, and inclusion for artists with IDD; (b) physical distancing mandates accelerated technology implementation; and (c) common technologies may reduce barriers and expand access, while also introducing new challenges. Specifically, we asked:

1. How did the purpose of technology change during the COVID-19 pandemic for community-based creative organizations that support artists with IDD?

- How and why did these organizations adapt their programming and engagement practices?
- How did these adaptations affect the organizations’ efforts to provide connection, access, and inclusion for artists?
- What unexpected innovations and pedagogical changes emerged from rapid virtualization?
  2. How do these adaptations map onto the We-Making framework, and what do they suggest for equitable practice during- and post-pandemic?

## 2 Methods

### 2.1 Research design and research team

This study utilized a quantitative-qualitative sequential design (21). First, we deployed an online survey gathering information on technology use in creative practices and pandemic adaptations. Second, informed by survey results, we conducted semi-structured interviews for more nuanced information about technology transformation and organizational impact.

The research team consists of three researchers with doctoral-level mixed-methodology training and varied field experience working with people with IDD. Our team takes a critical constructivist approach to qualitative data collection and analysis (22), drawing from prior both personal and professional experiences working with people with cognitive and physical disabilities. The first author has over 25 years of field experience facilitating expressive art projects with adults with IDD and their families and over 20 years of community-based social/behavioral science research expertise, working with community members with cognitive disability and their family caregivers. The second author brings 35 years of experience as an occupational therapist and health researcher; she and her three adult children identify as neurodivergent. The third author’s experience includes creating theatrical and choral performances with people of all ages with various disabilities, and research in inclusive postsecondary education for students with IDD.

### 2.2 Recruitment and data collection

For the survey participant recruitment, we used convenience sampling, contacting potential participants through national professional networks, manual web searches, and a publicly available list from the Vanderbilt Kennedy Center for Excellence in Developmental Disabilities. We distributed recruitment materials through email lists, websites, and social media platforms. For the interview participants, we used snowball sampling, informing survey participants about an additional interview opportunity via a follow-up email. To reach more potential interview participants, we also asked interviewees to forward our recruitment email to colleagues who have direct experience working with artists with IDD on technology implementation and activity adaptations. Participants provided informed online consent for the survey and verbal consent for interview participation.

Surveys were administered between July 2020 and December 2023 via REDCap, a secure, HIPAA-compliant web-based platform. Survey items were developed based on the literature and the research questions and included multiple-choice and open-ended items assessing technology use before and during COVID-19. The multiple-choice section included questions about organizational demographics (e.g., targeted audience group, age, race, ethnicity, and minority status), program settings (i.e., community, higher education, public school, clinical). The main survey questions asked about types of technology used (e.g., tablet, computer, 3-D printer) and the purposes of technology use before and during the pandemic, including (a) creativity, (b) connection, (c) access, (d) visibility, (e) inclusion, and (f) other (an open text response). In the open-ended questions, participants were asked to elaborate on their multiple-choice answers regarding the purpose of the technology use and provide additional insights and the changes they made during the pandemic.

Semi-structured interviews were conducted between August 2021 and December 2023. Preliminary survey findings informed the interview guide, which focused on the organizational processes and experiences transitioning to virtual programming. These structured questions include: (1) What steps did you take to make changes during the COVID-19 pandemic?; (2) How did you adapt your approaches to creative program activities and art making during the move to a virtual platform? (3) What barriers and challenges did you face? (4) Were there any silver linings? and (5) What changes will you carry forward?

### 2.3 Analysis

Survey data were analyzed using descriptive statistics to summarize organizational characteristics, technology purposes, and reported programming changes during the COVID-19 pandemic. We calculated frequencies and percentages for closed-ended items and reviewed open-ended survey responses to contextualize patterns in technology use and adaptation strategies. Interview transcripts and researchers’ reflective notes were deidentified and managed in VERBI Software (23). Analysis proceeded in two stages.

First, we conducted a structured thematic analysis to identify recurrent patterns across interviews, following the six phases described by Braun and Clarke (24) and procedures consistent with Nowell et al. (25). Three analysts independently familiarized themselves with the dataset through repeated reading, reflecting, and documenting, and then deductively coded the full set of transcripts in MAXQDA using an initial code set guided by the research questions while remaining open to inductive insights documented in analytic notes. Codes were iteratively compared and refined through weekly team meetings among the three researchers, resulting in a shared codebook with categories, subcategories, and definitions. After each coder re-coded the dataset, using the refined codebook, we resolved discrepancies through discussion and consensus; initial inter-rater agreement before consensus discussions was 97.5%, with most differences occurring at the subcategory level. We then identified key themes by reviewing coded extracts across participants to identify coherent patterns and relationships among codes. Then, the researchers finalized these themes through iterative coded narrative reviews and team discussions. Researchers’ reflections and analytic descriptions of coding and theme development were maintained in analytic notes to support transparency and rigor.

Second, we used reflexive thematic analysis, adapted from Braun and Clarke (26), to support deeper interpretation within and across themes. During theme development in the first stage, the team noted conceptual parallels with the We-Making Framework (19). Overwhelmingly, the COVID-19 pandemic-related changes occurred centering around the social connections, relationships, and solving shared challenges–social cohesion. Therefore, we used the framework as an interpretive lens to examine relationships among categories, underlying meanings, and contextual influences; the how and why these changes occurred in certain circumstances, while maintaining a reflexive and iterative analytic process. In this reflective process, researchers drew not only on the interviewers’ observations, capturing participants’ insights during the interview, but also on their own professional reflections in the field. Theme-related excerpts were re-examined in relation to relevant We-Making constructs. Throughout this stage, we moved repeatedly between coded extracts and the full dataset to ensure themes remained grounded in participants’ accounts.

## 3 Results

The survey data provided descriptive information about changes in the purpose of technology use, while the interview data offered insights into the roles of technology during the COVID-19 pandemic.

### 3.1 Participants

Eligible participants were representatives of creative arts organizations serving artists with IDD who: (a) had been involved for 1 year or more; (b) understood the mission, operations, and activities; (c) had substantial influence on programmatic decisions; and (d) understood the motivation of artists’ participation in the organization.

Thirty-seven participants from 33 organizations enrolled: 28 completed surveys and 25 completed interviews, with 16 completing both. The survey participants varied in organizational leadership roles, including program directors (*n* = 15), program managers (*n* = 7), and other roles (*n* = 6, i.e., board member, president, faculty member, therapist, social worker, or co-founder) for the survey. The interview participants expanded their roles in more direct encounters with artists, including program directors (*n* = 10), program managers (*n* = 9), lead direct care professionals (*n* = 4), and other roles (*n* = 2, i.e., therapist and social worker). All of the participants in leadership roles we interviewed had direct knowledge of both the programs and artists attending. Based on the survey responses, most participants represented visual arts groups (*n* = 21) or performing arts groups (*n* = 11), with two organizations including both. All organizations served artists with IDD (*n* = 28), with some also serving other disability populations: mental illness (*n* = 14), physical disabilities (*n* = 12), sensory disabilities (*n* = 8, i.e., deaf, blind, hard-of-hearing), and brain injury (*n* = 2), plus artists without disabilities (*n* = 8). Participating organizations served adults ages 26 + (*n* = 26), young adults ages 19–26 (*n* = 25), teens ages 12–18 (*n* = 12), and/or children under 12 (*n* = 10).

### 3.2 Purpose of technology use

Before the COVID-19 pandemic, organizations reported that technology was equally used for promoting creativity (96%), connection (96%), visibility (96%), inclusion (93%), and access (93%). During the pandemic, newly implemented technology was primarily aimed at connection (96%) and access (96%), with secondary purposes of inclusion (79%) and visibility (63%). Creativity (46%) ranked lower in their priorities during the pandemic.

### 3.3 Roles of technology in the COVID-19 pandemic

Our analysis identified four major themes regarding technology’s role:

#### 3.3.1 Theme 1: a platform for social connection

Organizations’ initial reaction was to find ways to communicate with isolated artists using phone calls, social media, and emails. Many implemented web-based platforms like Zoom and Microsoft Teams. Organizations initially pivoted away from art skills and production-oriented instruction, focusing instead on connectivity. Participants reported artists wanted “to be with their friends” (participant #23, female, director, performing arts organization) and “continue community for everyone” (participant #21, female, lead direct care professional, visual arts organization). Organizations realized they needed new ways to engage artists in two-dimensional online environments. Many developed routines to facilitate engagement: “When we get together… we have an opening ritual, something that immediately engages people, followed by activities like dance parties and virtual field trips” (participant #17, female, director, performing arts organization).

Virtual space offered new opportunities for artists to know each other as each shared and interacted with the whole group. One participant noted: “The biggest outcome for me was getting to know our actors better… with Zoom you really get to focus in on each person” (participant #23, female, director, performing arts organization) It expanded friendships: “We found surprisingly that some artists who never really initiated talking to one another in the physical studio are much more inclined to start a conversation on the Zoom interface” (participant #4, female, director, visual arts organization). These opportunities fostered deeper relationships: “I think the biggest value… is just that they want to be respected as human beings… as human beings who are artists” (participant #17, female, director, performing arts organization).

However, many participants commonly noted that a small number of their artists struggled to make connections and transfer creative practice online.

#### 3.3.2 Theme 2: a temporary and suboptimal solution

Organizations whose identity was built on in-person group dynamics initially viewed virtual space as temporary while awaiting a return to in-person practice. For these organizations, the unspoken energy of simultaneous group interactions and spontaneous encounters in a physical space served as the primary source of creative process and inspiration. These aspects were identified as limitations in two-dimensional environments. As one participant noted: “the physical studio versus the virtual, you. have the flat surface and you do not have the visual cues or things that stimulate artists” (participant #5, male, manager, visual arts organization). Additionally, without having end goals like performances or exhibitions, long-term motivation in the virtual space was challenging.

These organizations often simplified practices rather than creating innovations. One noted: “I do not think we have maximized the technology, but then it gets complicated, so we try to keep it really simple.” Choral groups had limited rehearsals due to connection delays: “They had to troubleshoot. having everyone mute themselves except for the pianist due to sound delays” (participant #19, female, director, performing arts organization). They restricted repertoires: “Some songs are too difficult to do online. I eliminated those” (participant #19, female, director, performing arts organization).

Organizations also faced common challenges with limited space and materials for at-home artists, restricting artistic expression, collaboration, and products. A theater company highlighted challenges with full-cast engagement: “you cannot have big groups in Zoom, so we had to learn breakout sessions… and still keep things intimate” (participant #23, female, director, performing arts organization). These organizations felt they lost physical space and ambience, promoting “model learning” where artists see “other peer artists making their own work” (participant #8, male, manager, visual arts organization), which is a necessary formula for working as independent artists in shared physical space.

However, all mentioned benefits regarding accessibility: “Hybrid activities allow singers who moved away to still participate, as well as those for whom the commute was too long” (participant #19, female, director, performing arts organization).

#### 3.3.3 Theme 3: a catalyst for innovative strategies

Several organizations leveraged virtual/digital platforms to enhance missions and expand reach, discovering promising practices for long-term change by increasing access, exposure, and inclusion: “The biggest, most amazing beneficial thing was going virtually, which I definitely think is something we are going to keep bringing to the future” (participant #3, female, director, performing arts organization). These organizations often exhibited shifts in leadership, programming direction, or institutional support before or during the pandemic, making them more open to imagining technology’s potential innovative approach rather than merely replacing existing activities.

One participant highlighted increased access: “We were able to have guest teachers from the UK and other U. S. cities without needing to pay for accommodations, which is huge” (participant #3, female, director, performing arts organization). Another described: “When Covid hit, we had to close down. That is when we developed our virtual program with 50 different classes” (participant #9, female, director, visual arts/performing arts organization). Visual art studios noted virtual engagement provided opportunities to incorporate learning materials and foster connections through online resources and networking: “We actually launched a Zoom group when the pandemic happened… to bounce ideas around and build up that network” (participant #5, male, manager, visual arts organization).

Other innovative strategies increased artists’ access to creativity and expanded inclusive practices. Some organizations shared how leveraging technology expanded reach beyond their disability network to general community organizations. A visual art gallery used social media to forge partnerships with political groups and community art organizations, leading to increased digital art commissions and sales. This led to a large national project: “We were contacted by the Biden campaign. They licensed a piece of his work… and the artist actually got to meet the president over Zoom” (participant #7, female, co-founder, visual arts organization). The same organization described how mutual interaction can also be implemented via virtual space, building upon prior non-hierarchical social relationships among artists with and without disabilities:

Before the pandemic, it would be people with and without disabilities in the same shared space; there wasn’t like this weird hierarchical power dynamic. It wasn’t a mentor-mentee, it wasn’t teacher-student… So we did corresponding virtual events. it was artists and non-artists, people with and without disabilities, just hanging out virtually (participant #7, female, co-founder, visual arts organization).

A performing arts organization collaborated with an external digital production company on multimedia projects, diversifying artistic format and broadening audience reach, creating an inclusive art network. Participants noted “these positive things through technology are here to stay… because of this virtual network, more integration can happen in good ways. In the virtual class, we have general public folks taking art classes together with disabled artists” (participant #23, female, director, performing arts organization).

#### 3.3.4 Theme 4: a driver of pedagogical change

Some organizations noted significant pedagogical shifts toward participant-responsive practice, triggered by new virtual spaces. Flat screens provided a two-dimensional space with equal visibility and opportunity for all participants, regardless of their engagement style. In this equally divided virtual space, instructors became more adept at noticing subtle verbal and non-verbal cues easily unnoticed in an in-person space; they received real-time feedback from all: “It is important what you are in tune with their needs and what they are interested in, excitement, and connection for the moment” (participant #1, female, therapist, visual arts organization). This fostered participant-driven approaches: “through Zoom we were able to be even more creative and find ways to connect with each other even more, and learn how to notice micro movements, responses and energy” (participant #11, female, president, performing arts organization). They recognized first-time artists’ “creative genius “[previously unrecognized]: “We’ve been told. that he’s going to generally play background roles because of language barriers and other challenges [imposed assumptions]. What we found on Zoom was some of those challenges were just not there” (participant #17, female, director, performing arts organization).

Visual cues and information sharing became prominent on two-dimensional platforms, supporting equitable access and visibility regardless of verbal communication and physical movement capacities, particularly for artists with limited opportunities in physical spaces. Some organizations shared that close-up capacity of virtual learning promoted co-facilitation and co-construction pedagogy. One visual art studio noted: “The art helps lubricate the social environment because artists can hold their artwork up, and you know be proud of it, without having to describe anything” (participant #12, female, social worker, arts organization). For example, one dance company shifted from choreographed to fully improvised productions, allowing freedom of expression for each dancer. Unexpected benefits included greater inclusion and visibility of dancers with limited mobility and greater trust between members. Greater visibility occurred because Zoom’s close-up picked up subtle, intentional, expressive movements. The director noted: “I think we have enough trust with each other, that a piece would just appear organically [rather than choreographed] now… even more creative… connect with each other” (participant #11, female, president, performing arts organization).

Organizations emphasized that this curricular and pedagogical shift was an ongoing process, not a one-time fix. Artists and instructors collaborated through trial and error through ongoing dialogues: “we found that we had to have those points of action, where it is not just us presenting… but opportunities for artists to say how they felt about what we were doing or what the challenge was” (participant #17, female, director, performing arts organization). This pedagogical shift led to long-term impacts for some organizations, enhancing effectiveness even as in-person activities resumed: “We will not go back to choreographed work anymore. because among our group the piece belongs to the group” (participant #11, female, president, performing arts organization).

### 3.4 Meaning making through the we-making framework

As we listened to the voices of the art studio representatives during our in-depth interviews and again during our thematic analysis, it became clear that what we were hearing was the focus on shared time, space, and technology for social cohesion, similar to the We-Making Framework. Through the lens of the We-Making Framework, we then explored the deeper meaning of the change that the COVID-19 pandemic produced. The We-Making Framework provides the terminology to describe the social cohesion and related components and elements that impact the process for producing equitable change.

**Figure 2:**
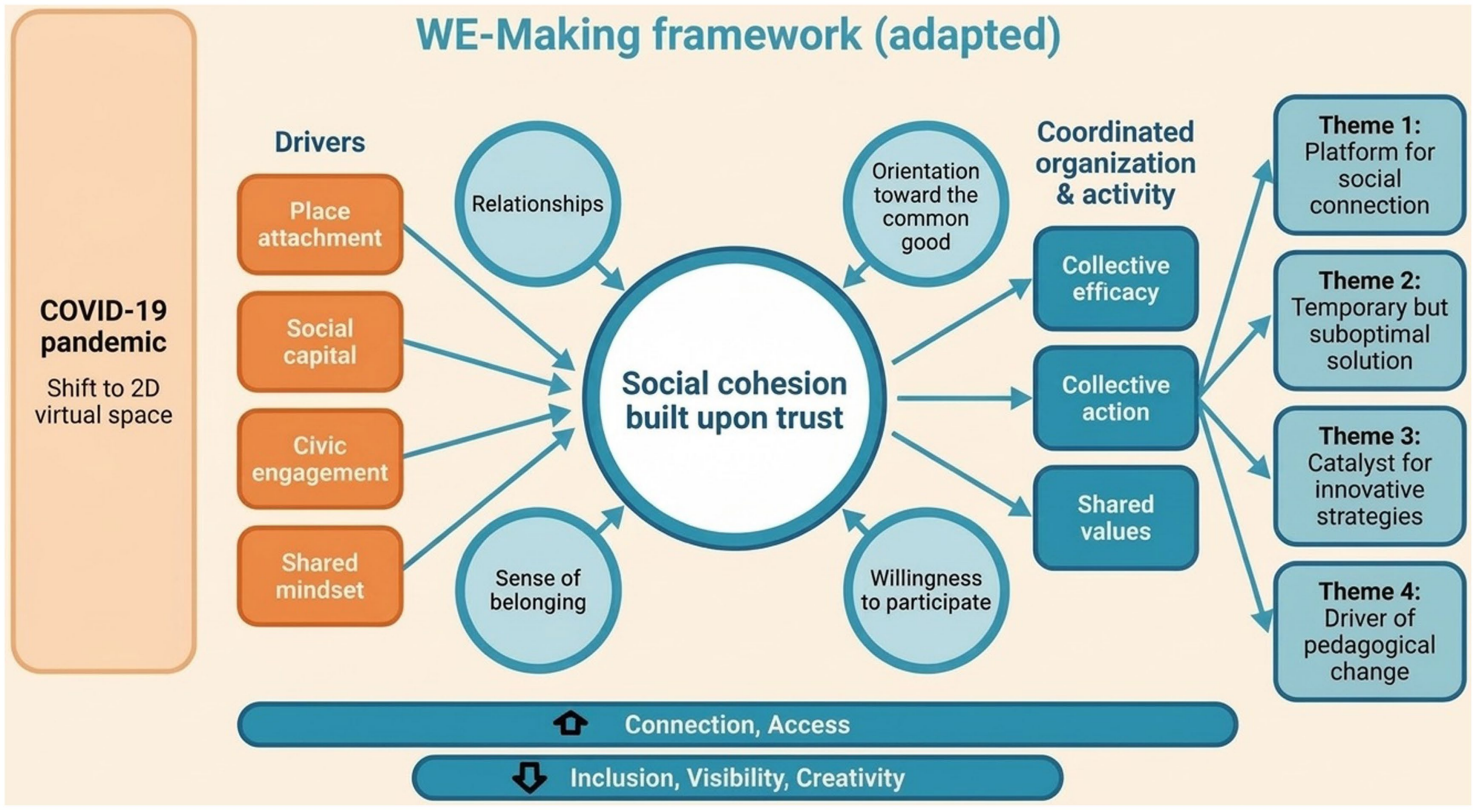
Examining how the COVID-19 pandemic transformed creative organizations in virtual space through the We-Making framework. Adapted in part from the WE-Making conceptual framework (19). Adapted with permission. Source: https://metrisarts.com/wp-content/uploads/2021/04/we-making_literature-review.pdf.

The We-Making Framework defines social cohesion as when “individuals feel and act as part of a group oriented toward working together” (p. 21), grounded in five characteristics: trust, relationships, sense of belonging, willingness to participate, and orientation toward the common good for equitable change. These can be activated by drivers, including place attachment, social capital, civic engagement, and mindset. Please see the key components of the We-Making Framework (19) in relation to the four themes identified in Figure 2.

Our thematic analysis identified that during the pandemic, the newly implemented virtual space became crucial for “Theme 1: a platform for social connection*”* among almost all participating organizations. Due to the pandemic’s physical restrictions on accessing community social spaces, the virtual space provided by community-based art organizations became the sole avenue for social connection for most artists. This led these organizations to adapt their activities beyond their usual art-related pursuits, integrating opportunities for socialization and human connection. During the COVID-19 crisis, shared challenges among community artists acted as a catalyst for strengthening social cohesion through drivers in the We-Making framework: *place attachment*, *civil engagement*, *shared mindset*, and *social capital* (19).

When organizations pivoted from in-person locations to virtual spaces, their previously established *place attachment,* through the organizational membership, facilitated and supported this shift. Artists had already developed feelings of trust and a sense of belonging through arts and cultural activities (19). Although the physical place changed, the continued *place attachment* provided an environment that promoted social connections and “a sense of belonging to place and group” (19), p. 27. This enabled creative organizations to effectively maintain connectivity while coordinating virtual activities and making necessary adjustments to their programming.

Though not ideal, many embraced the two-dimensional virtual space, driven by the fundamental human need for interaction. As we observed in the theme 1-related participants’ narratives, this digital realm became a hub primarily for *civic engagement*, where members deepened connections and relationships. As we heard in their interviews, as artists and organization staff familiarized themselves with this new virtual environment, they recognized common struggles: social isolation, limited life control, fear of the unknown, and scarce resources and choices. The growing understanding and empathy among members steered them to work toward the common good. United by this shared *mindset*, many participants exchanged ideas and resources to address these collective needs, often reaching beyond individual needs, striving for *social capital*.

For many organizations, this shift toward collective good resulted not only in adding social activities but also in creating opportunities for lasting changes. Our thematic analysis identified these changes in “Theme 3: a catalyst for innovative strategies” and “Theme 4: a driver of pedagogical change.” The virtual space, with its two-dimensional and singular nature, fostered “*bonding capital*” (19), p. 29. In this digital realm, facilitators and artists enjoyed enhanced opportunities “to tell their own community stories and determine their own agenda for change” (19), p. 30. Ultimately, this shift led to a reevaluation and transformation of core artistic processes and equitable practices; what the We-Making Framework calls “equitable community well-being” (19), p. 41.

Many changes related to “Theme 3: a catalyst for innovative strategies” were observed through new innovations by “bridging social capital” (19), p. 31. By leveraging virtual space, many organizations extended their missions by accessing external resources and connecting with peer organizations, new partners, and audiences. This strategic move allowed them to tap into previously unreachable and unrealized networks, fostering collaborations and partnerships that transcended geographical limitations through an in-person-only practice. By doing so, they enriched their programming with diverse perspectives and resources. Furthermore, several organizations invented new strategies to help overcome limitations of synchronous virtual engagement. Our participants shared a few strategies in their interviews, including creating recorded sessions, utilizing and connecting to external online resources, and utilizing professional media production services. Such innovations not only offered opportunities for alternative creative expression but also enhanced *social capital through* visibility, accessibility, and inclusivity.

In theme 4, “a driver of pedagogical change,” creative processes underwent significant change influenced by an emphasis on participant-driven pedagogy, a concept parallel to “build and share power through community ownership” (19), p. 38 in the We-Making framework. This participant-responsive approach prioritizes community co-design, co-creation, and co-production, providing space for members to guide the process. This strengthened social cohesion and contributed to “working toward the common good,” promoting equitable change. A few examples from the interviews highlight lasting pedagogical changes. One visual art studio responsibly co-designed their virtual art practice with their artists in the digital space, and the co-facilitation of art sessions became a natural, effective format for their group. Similarly, one dance group embraced the close-up movement of each dancer. Member-informed choreography became the norm of their creative process over a director-driven approach.

## 4 Discussion

Consistent with prior work highlighting the importance of technology-enabled access and social time for mitigating pandemic-related social isolation and loneliness (7, 18), our findings indicate that community-based arts organizations serving artists with IDD used technology primarily to sustain connection and access during the COVID-19 pandemic. Survey results showed a clear shift in organizational priorities toward connection and access, with comparatively less emphasis on creativity, inclusion, and visibility. Interview data contextualized these patterns and identified four interrelated roles of technology: (1) a platform for social connection, (2) a temporary and often suboptimal substitute for in-person engagement, (3) a catalyst for innovative strategies, and (4) a driver of pedagogical change.

Across themes, the We-Making framework helped explain why some organizations were able to rapidly create viable virtual spaces. Existing relationships, trust, and a sense of belonging functioned as forms of place attachment that could be reconstituted online, allowing organizations to maintain social cohesion despite the loss of physical gathering spaces. At the same time, the constraints of two-dimensional platforms shaped participation and practice, particularly in organizations whose identity and pedagogy were strongly grounded in shared physical ambience, spontaneous interaction, and observational learning.

Notably, technology-mediated programming did not merely preserve services. In some organizations, it prompted reflective shifts toward more participant-responsive and power-sharing approaches, aligning with We-Making concepts related to collective efficacy, shared mindset, and building equitable community wellbeing. Virtual participation sometimes surfaced overlooked strengths and expanded opportunities for co-facilitation, co-production, and bridging social capital through new partnerships, audiences, and professional networks.

### 4.1 Limitations

This study has several limitations. First, convenience and snowball sampling likely favored organizations that were well-resourced, well-networked, and able to survive the transition to virtual or hybrid formats. The experiences of organizations that paused or closed operations may be underrepresented, limiting generalizability.

Second, data were drawn from organization representatives rather than artists with IDD, which restricts the interpretation of the lived experience and perceived quality of participation from artists’ perspectives. Including artists as co-researchers or primary participants in future studies would strengthen validity and alignment with inclusive research principles.

Third, the study captured adaptations during an evolving public health context. We were not able to systematically follow all organizations longitudinally to examine how virtual and hybrid practices were sustained, refined, or discontinued post-pandemic.

Finally, technology access, caregiver or staff support, and digital literacy varied across settings and were not measured in ways that would allow fine-grained analysis of how these factors shaped participation and outcomes.

### 4.2 Implications

Findings suggest that technology can support equitable participation when it is intentionally designed to advance connection, access, inclusion, creativity, and visibility, rather than being used solely as an emergency substitute for in-person programming. Virtual formats may be particularly beneficial for maintaining engagement for artists who face transportation barriers, health-related constraints, limited supports, or geographic distance.

However, virtual and hybrid programming should be implemented with explicit attention to what is gained and what is lost relative to embodied, place-based arts participation. Two-dimensional platforms may constrain implicit learning, shared sensory experiences, and spontaneous group dynamics that support modeling, collaboration, and artistic motivation.

For post-pandemic practice, organizations may benefit from structured decision-making that clarifies:

*Membership and power*: Who participates and who holds power to make decisions about the creative process, facilitation, and ownership.

*Purpose*: Whether the primary aim is social connection, creative skill-building, production, community visibility, inclusion, or a combination.

*Role of technology*: Whether technology is used to extend existing communities, build new communities, support asynchronous creativity, increase accessibility, or broaden audiences and partnerships.

Operationally, these considerations point to the need for investment in accessible platforms, training for artists and staff, support for caregivers and direct support professionals, and practices that center choice and individual preferences.

## 5 Conclusion

Community-based arts organizations serving artists with IDD played a critical role in sustaining social connection and community participation during the COVID-19 pandemic. Technology adoption shifted organizational priorities toward connection and access, while simultaneously exposing persistent inequities in resources, digital access, and the quality of participation.

Interpreted through the We-Making framework, the effective transition to virtual space was supported by pre-existing social cohesion and place attachment within organizations, which enabled communities to reconstitute “shared space” online. Although virtual environments could not fully replicate the relational and embodied qualities of in-person arts practice, they also created conditions for meaningful innovation, including expanded networks, increased accessibility for some participants, and pedagogical shifts toward more participant-responsive and power-sharing approaches.

Together, these findings underscore the importance of treating community arts organizations as public health–relevant partners and investing in inclusive, accessible, and flexible models of arts participation that can support community wellbeing during crises and beyond, particularly for these under-resourced and underserved communities who face inequitable access.

## Data Availability

The raw data supporting the conclusions of this article will be made available by the authors, without undue reservation.
